# Innovative Management of Blood Culture-Negative Endocarditis With Pulmonary Septic Emboli in a Patient With a Biventricular Pacemaker and Implantable Cardioverter Defibrillator (BiV-ICD) and Psychosocial Stressors: A Case Report

**DOI:** 10.7759/cureus.65116

**Published:** 2024-07-22

**Authors:** Sabina P Coica, Kyla N Wilson, Bassam Baroudi

**Affiliations:** 1 Cardiology, William Carey University College of Osteopathic Medicine, Hattiesburg, USA; 2 Internal Medicine, William Carey University College of Osteopathic Medicine, Hattiesburg , USA; 3 Cardiology, Memorial Hospital at Gulfport, Gulfport, USA

**Keywords:** implantable cardioverter-defibrillator, biventricular pacemaker, staphylococcus epidermidis, psychosocial factors, pulmonary septic emboli, blood culture-negative endocarditis

## Abstract

Blood culture-negative endocarditis (BCNE) poses significant diagnostic and therapeutic challenges and is associated with notable morbidity and mortality. When presented concurrently with other comorbidities, these challenges and the chances of morbidity and mortality significantly increase. This case presents right-sided BCNE accompanied by pulmonary cavitary lesions in a patient with a history of supraventricular tachycardias (SVT), a biventricular pacemaker and implantable cardioverter-defibrillator (BiV-ICD), alcohol use, and anticoagulant noncompliance. The patient missed follow-up appointments for six months after the death of his wife, leading to increased alcohol use and noncompliance with medications. During this period, his home monitoring device was offline. Once reconnected, it detected several episodes of SVT and ventricular tachycardia (VT), prompting a wellness check. He presented to the cardiology clinic with shortness of breath and a cough producing brown-tinged sputum. Evaluation revealed cavitary lesions in the lingula and left lower lobe, a vegetation on his tricuspid valve, and vegetations on his endocardial leads, despite negative blood cultures. Tuberculosis testing was negative, while sputum cultures were positive for Haemophilus influenzae. After ruling out other possible infectious causes of the cavitary lesions, septic emboli were suspected as the cause. Broad-spectrum antibiotics were begun and surgical intervention was done to replace the tricuspid valve and remove the endocardial leads. This procedure was complicated by fibrosis of the leads at the coronary sinus, necessitating their cutting at the superior vena cava and leaving them inside the patient until laser therapy could be performed for their removal. The patient's history of bradycardia and SVTs required the ongoing use of a pacemaker. Inventory discrepancy during the placement of the new pacemaker epicardial leads lead to complications warranting an alternative approach to lead implantation. A traditionally used epicardial lead was placed on the right ventricle for pacing, and an innovative technique was employed to place an endocardial lead on the right atrium epicardium for sensing. This case underscores the importance of thorough evaluation and collaborative management strategies to optimize outcomes for patients with concomitant cardiac and pulmonary pathologies, particularly in the context of underlying psychosocial stressors. Additionally, it demonstrates solutions to challenges that can arise during surgery and presents an alternative lead placement technique for physicians who have only one epicardial lead available after removing infected endocardial leads. This is illustrated by the innovative use of an endocardial lead as an epicardial sensing lead.

## Introduction

Microbial infection of the heart’s endothelial lining is defined as infectious endocarditis (IE). Clinically, it presents with fever, malaise, tachypnea, and pathologic cardiac changes such as heart murmur development or progression. Diagnosis of IE is commonly assessed with the 2023 Duke-International Society for Cardiovascular Infectious Diseases (ISCVID) criteria [1]. The two major criteria include positive blood cultures and endocardial involvement in echocardiography. IE is further defined by the location of the infection and the structures involved [2]. Right-sided IE refers to the tricuspid and pulmonic valve and only accounts for 5-10% of cases. It is associated with IV drug use, intracardiac devices, and central venous catheters [2]. IE is caused by the seeding of these structures and is associated with a one-year mortality of 30% [1]. There is insufficient data published on the diagnosis, features, and management of right-sided IE compared to left-sided IE. 

The introduction of BCNE into a clinical presentation of IE exacerbates the challenges, presenting a scenario with limited guidance on appropriate management strategies. BCNE occurs in the range of 10-20% of IE cases and is associated with increased negative outcomes compared to culture-positive endocarditis [3]. BCNE requires further steps to discover the perpetrating infective organisms such as open chest surgery to remove and culture the vegetation.

An example of an intracardiac device that can cause IE is a biventricular pacemaker and implantable cardioverter-defibrillator (BiV-ICD). This device is used in heart failure patients, featuring leads connected to a pulse generator typically implanted subcutaneously in the left anterior chest [4]. This device includes a right ventricular defibrillator lead and a left ventricular pacing lead located in the coronary sinus [4]. An atrial pacing lead may also be used if the patient requires it for conditions such as atrial fibrillation or bradycardia [4]. In the presence of ventricular arrhythmias, the BiV-ICD recognizes the abnormal rhythm and addresses it with anti-tachycardia pacing or defibrillating shocks while also sending a notification to the cardiology clinic via remote monitoring [4]. In the situation where infection of this device ensues and open chest surgery is required for device removal, two epicardial leads are typically placed, one on the right atrium and one on the right ventricle. This ensures that the patient is still receiving the pacing they need [5]. 

A severe complication of IE is septic embolization. Septic pulmonary embolism is a rare disorder caused by right-sided IE with many manifestations and causes. Due to the similar presentation of pneumonia, the diagnosis can be missed, thus influencing prognosis [6]. Within days, septic emboli can rapidly develop into cavitary lesions [7]. The presence of cavitary lung lesions poses an additional challenge, exacerbating the morbidity and mortality risk in a patient concurrently diagnosed with BCNE [6]. Broad-spectrum antibiotics should be started promptly and other infectious causes such as tuberculosis should be ruled out [6].

In lieu of the medical complications addressed, psychosocial factors are pertinent in cases of IE. The experience of losing a spouse commonly brings about feelings of depression, loneliness, sadness, and a loss of hope for the future [8]. This can often lead to neglect of the patient's own health and noncompliance with medications, exacerbating their condition and complicating treatment outcomes [9]. Additionally, this grief often leads to heightened smoking and alcohol misuse, especially among men [9]. Alcoholism can play a crucial role in many health conditions, the heart being one of the organs that can be affected. Alcoholism is a recognized risk factor for right-sided IE, further complicating the clinical picture and emphasizing the importance of comprehensive management approaches [2].

## Case presentation

Patient information

A 58-year-old man missed follow-up appointments for six months after the death of his wife. During this time, his home monitoring device, connected to his BiV-ICD, was offline. Upon reconnection, remote monitoring detected several episodes of supraventricular tachycardia (SVT) and ventricular tachycardia (VT), prompting a wellness check. He was instructed to visit the cardiology clinic as soon as possible. When he presented to the clinic the next day, he appeared cachectic and reported shortness of breath, a cough producing brown sputum, and night sweats that had persisted for three months. He shared that he had stopped taking his anticoagulation medication and began drinking alcohol after his wife passed away six months prior. Over the past six months, he had also lost 40 pounds and developed a new chronic cough. At the time of his presentation, he had ceased drinking alcohol. His past medical history included congestive heart failure, chronic obstructive pulmonary disease (COPD), hypertension, hyperlipidemia, a BiV-ICD, and tobacco and alcohol use. Due to his symptoms and medical history, the patient was admitted to the hospital for further evaluation and management.

Clinical findings and diagnostic assessment

On physical examination, the patient was alert and oriented but appeared cachectic. His heart had a regular rate and rhythm with a pansystolic murmur over the left sternal border. An audible stridor was noted, along with diminished breath sounds at the base of the left lung. There was no edema or jugular venous distension. The abdomen was soft, non-distended, and non-tender.

Diagnostic methods included computed tomography (CT) of the chest, blood cultures, sputum culture, transthoracic echocardiogram (TTE), transesophageal echocardiogram (TEE), and heart catheterization.

**Figure 1:**
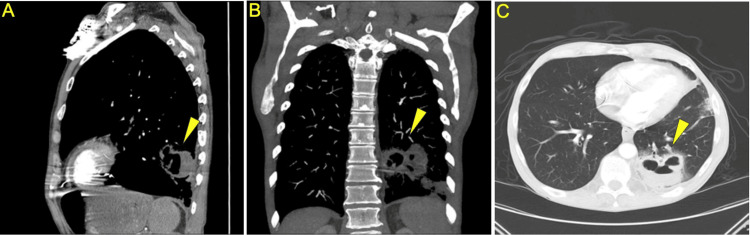
Cavitary lesion in left lower lobe. (A) Sagittal CT angiogram chest with contrast. (B) Coronal CT angiogram chest with contrast. (C) Apex to base CT angiogram chest with contrast.

A CT angiogram with contrast of the chest was performed due to the patient's pulmonary symptoms. This revealed cavitary lesions in the lingula and left lower lobe, suggesting a possible pulmonary abscess (Figure 1). Differential diagnoses included septic emboli, cavitary pulmonary infarction, aspiration pneumonia, and pulmonary tuberculosis. Tuberculosis testing, conducted by the Infectious Disease team, yielded negative results. Blood cultures showed no growth after three days, but sputum culture was positive for *Haemophilus influenzae*. 

**Figure 2:**
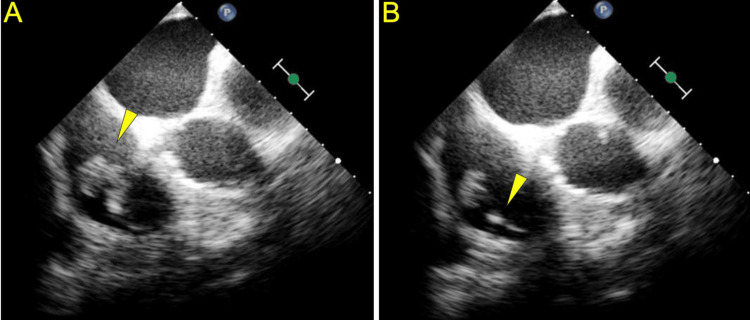
Transesophageal echocardiogram (A) Vegetation on the septal tricuspid leaflet. (B) Vegetation on the pacemaker lead in the right ventricle.

Due to the patient's new murmur on cardiac auscultation, a TTE was performed to further investigate the cause. The TTE suggested the presence of tricuspid valve vegetation. This was confirmed by a TEE, which revealed a large vegetation on the septal tricuspid leaflet involving the BiV-ICD lead (Figure 2). Following the TEE results, the differential diagnosis for pulmonary manifestations shifted towards septic emboli as the most likely cause. Cardiothoracic surgery was consulted to remove the vegetation and requested a heart catheterization prior to proceeding with the surgery. The heart catheterization revealed that the lateral coronary sinus branch was nearly occluded at the site of the BiV-ICD lead, indicating potential difficulties in removing and replacing the lead.

Considering the complex clinical and diagnostic presentation observed in this case, the prognosis initially seemed unfavorable. Nonetheless, expedited surgical intervention and the administration of appropriate medications provided a potential avenue for improvement in the patient's condition.

Therapeutic interventions

Once hospitalized, the patient was restarted on his previous medications which included atorvastatin 40 mg, Coreg 12.5 mg, Zetia 10 mg, lisinopril 10 mg, and Protonix 40 mg. Broad-spectrum antibiotics, Unasyn 3 g IV every six hours and moxifloxacin 400 mg oral (orally) daily, were first prescribed for the pulmonary cavitary lesions and Rocephin 2 g IV every two hours was added once sputum cultures revealed *H. influenzae*. 

**Figure 3:**
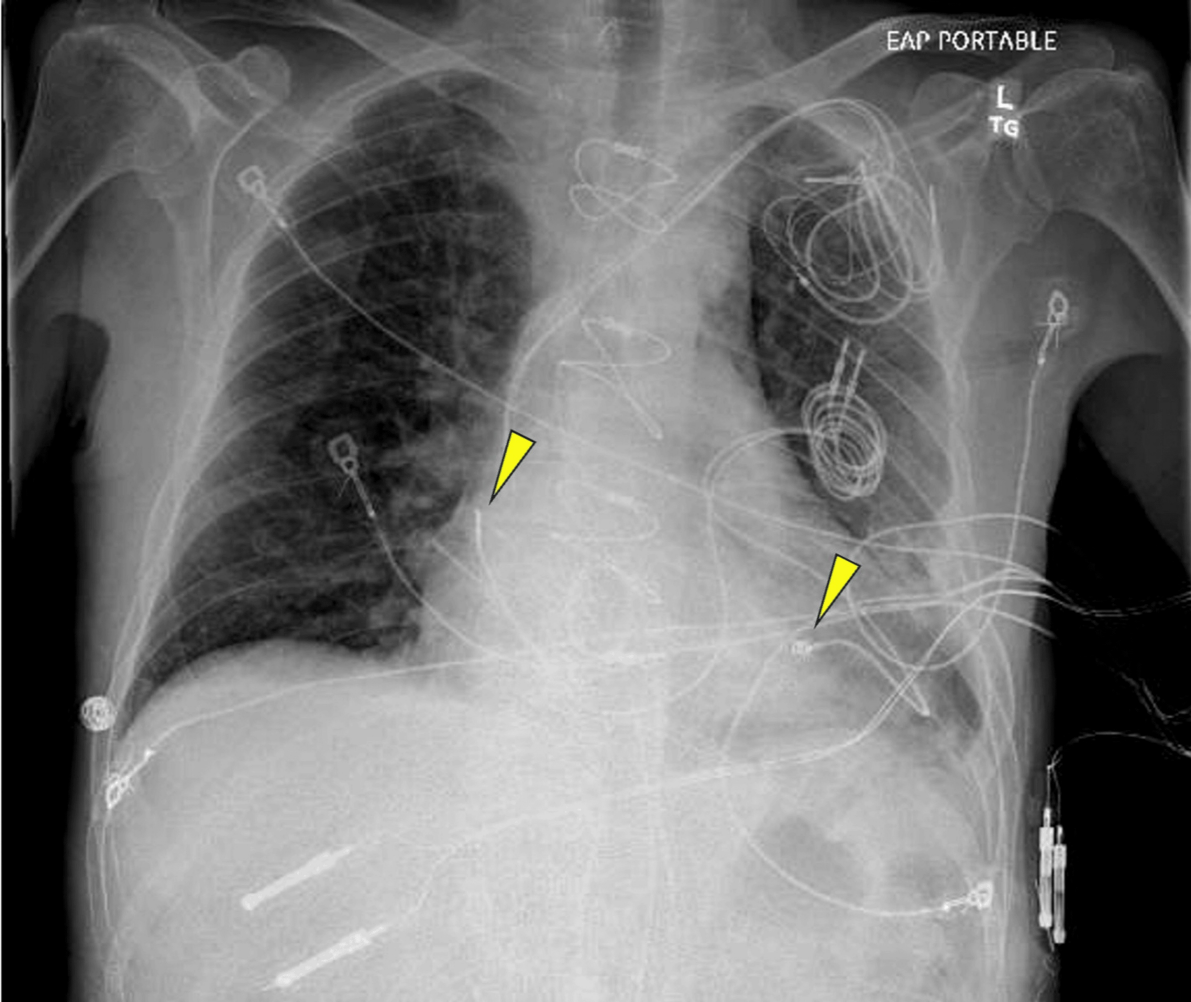
X-ray imaging of the endocardial lead placed on the epicardium of the right atrium (left arrow) and an epicardial lead placed on the surface of the right ventricle (right arrow).

Cardiothoracic surgery was performed, during which the tricuspid valve was replaced with a 29 mm Edwards tissue valve. The atrial, right ventricle, and right coronary sinus leads were cut and removed at the superior vena cava junction. The BiV-AICD was also removed, and attempts were made to extract the leads. However, the leads were firmly fixed and could not be dislodged from the myocardial tissue, indicating a need for future intervention with laser therapy. Given the patient's ongoing need for pacemaker intervention, two epicardial leads were placed: one on the right ventricle and the other on the right atrium, both connected to a temporary external pacemaker. Additionally, a permanent epicardial lead was placed on the right ventricle. Since another epicardial lead was unavailable, an innovative strategy of placing an endocardial lead on the surface of the right atrium was implemented. However, this lead could only be used for sensing rather than pacing (Figure 3). These leads were tunneled under the skin into the left lateral chest wall and monitored and tested in the intensive care unit (ICU).

**Figure 4:**
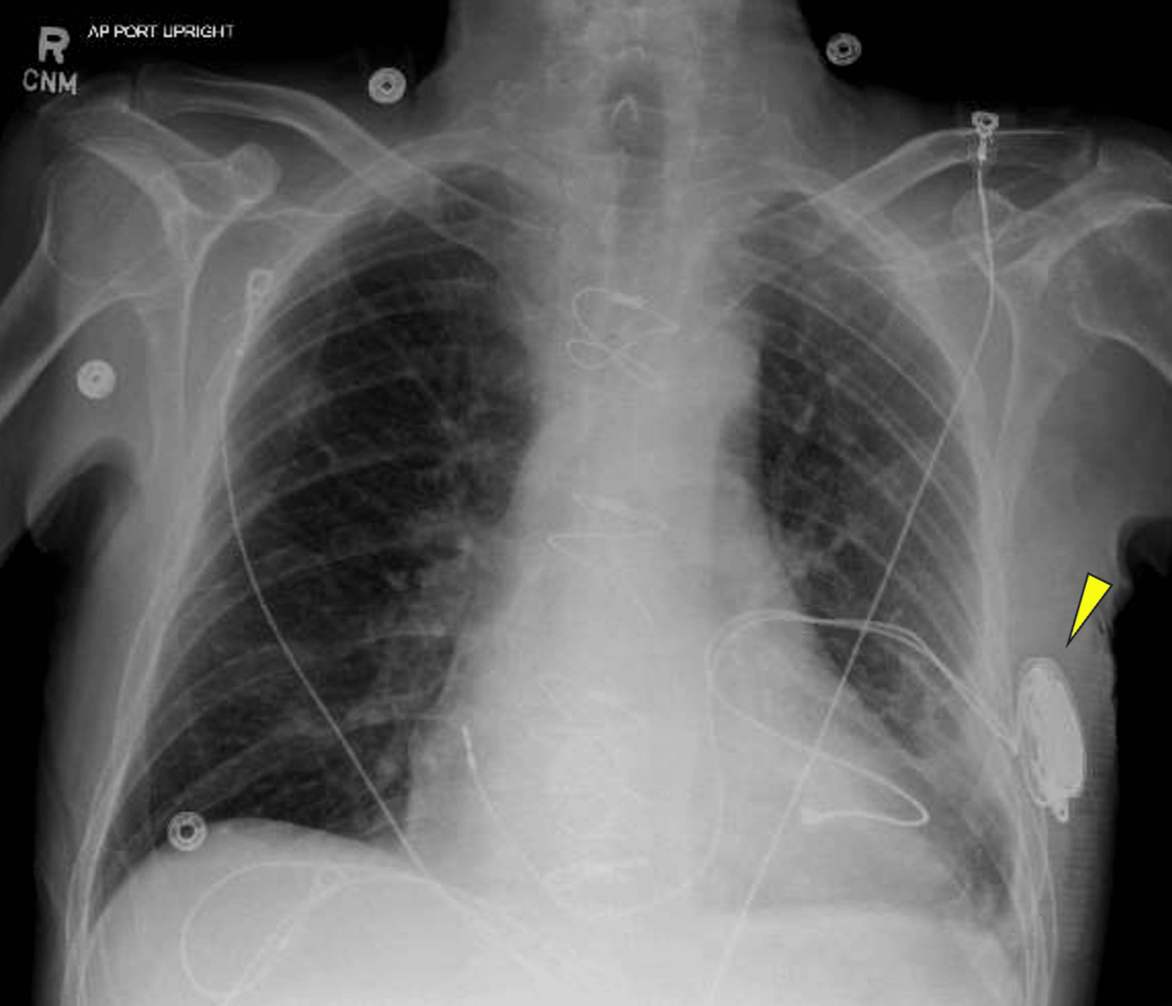
X-ray of the newly placed leads on the epicardium to a new pacemaker beneath the left axilla, away from the original pocket.

Seven days later, laser therapy was successfully performed to remove the previous BiV-ICD leads from the left subclavian vein and the superior vena cava. During this procedure, the temporary pacemaker epicardial leads were removed and the right ventricular and right atrial permanent leads that had been placed on the epicardium in the prior surgery were attached to a new pacemaker. This permanent pacemaker was implanted inferior to the left axilla, away from the original pacemaker pocket (Figure 4). Cultures of the vegetation from the tricuspid leaflet grew *Staphylococcus epidermidis*. Rocephin was prescribed to continue for six weeks post-operatively. Additionally, double coverage was achieved with rifampin 300 mg orally twice daily for six weeks. Lifelong suppression with doxycycline was recommended to begin after the initial six weeks of treatment.

Follow-up and outcomes

After being discharged from the hospital, the patient received instructions to complete a six-week course of antibiotics, followed by lifelong therapy with doxycycline. Once the patient has healed, future interventions include placing a new defibrillator system and connecting it to the patient’s right chest, away from the original pocket. He was scheduled for regular follow-up appointments with the cardiology team to monitor the function of the pacemaker, and a repeat chest x-ray was planned to monitor the resolution of septic emboli and pneumonia. Additionally, the patient was counseled on the importance of medication adherence and the risks associated with discontinuing medications, as well as the benefits of quitting alcohol and tobacco. He demonstrated comprehension of these instructions, and he expressed a willingness to actively participate in managing his health.

## Discussion

IE is an intricate condition, capable of affecting numerous bodily systems. Patients with cardiac devices face heightened susceptibility to this ailment. The modified 2023 Duke-ISCVID criteria serves as the cornerstone for diagnosing IE, yet the presence of BCNE adds another layer of complexity [10]. These variants challenge the reliance on positive blood culture results in the modified 2023 Duke-ISCVID criteria and rely on the presence of vegetations discovered on echocardiogram. A multidisciplinary approach is imperative for managing any case of IE. However, in instances of BCNE, it is crucial for ensuring comprehensive evaluation and tailored treatment plans [10].

This case involved a patient with a BiV-ICD, a history of alcohol use, and pulmonary manifestations. His symptoms which included fever, night sweats, and a new cardiac murmur heard during a physical exam, as well as pulmonary cavitations on chest CT, raised suspicion of IE and vegetations in the heart. These were confirmed by TTE and TEE. Prompt cardiothoracic surgery was scheduled, but preoperative heart catheterization revealed that the coronary sinus branch was nearly occluded, predicting complications.

During surgery, the leads were found to be fibrosed and were cut at the superior vena cava junction. Laser therapy was necessary to remove the remaining leads. Additional challenges arose when only one epicardial lead was available. Typically, two epicardial leads are placed for pacing, but this was not feasible. The surgeon implemented an innovative strategy not documented in the literature: an endocardial lead, usually not used on the outer surface of the heart, was placed on the epicardium of the right atrium for sensing. This adjustment was tested in the ICU and proved effective for the patient's pacing needs. This novel approach offers a potential solution for other physicians facing similar challenges during surgery. However, this approach should only be used when two epicardial leads are not available, as it presents limitations to the pacemaker's functionality. Specifically, the atrial lead is used for sensing rather than pacing. 

The patient's initial pulmonary symptoms, including a productive cough with brown sputum, led to the discovery of pulmonary cavitations on chest CT. Following guidelines, broad-spectrum antibiotics were promptly initiated. Further testing was conducted to rule out infectious causes; tuberculosis testing was negative, and sputum cultures revealed *H. influenzae*. The antibiotic regimen was adjusted accordingly. However, since *H. influenzae* does not typically cause pulmonary cavitations, a more likely diagnosis was pulmonary septic emboli resulting from cardiac vegetation. Similar presentations of BCNE can be compared to this case to help reach a diagnosis promptly. However, it remains crucial to rule out other potential causes and avoid assumptions.

The patient's previous noncompliance with essential anticoagulant medications and increased alcohol intake were crucial factors in his presentation. Adherence to anticoagulant therapy might have reduced the likelihood of heart valve vegetation fragments dislodging, which led to pulmonary septic emboli. Additionally, psychosocial factors played a significant role in his condition. The loss of his wife profoundly impacted him, leading to increased alcohol consumption and neglect of his well-being, causing him to ignore preexisting symptoms. It is essential for physicians to recognize and address the challenges patients face in their lives that may contribute to health neglect. This patient was fortunate to receive the necessary care in time, but others may not be identified until it is too late. 

The multidisciplinary approach in this case, involving cardiology, cardiothoracic surgery, and infectious disease specialists, facilitated a comprehensive evaluation and tailored management strategies. Prompt recognition of the patient's complex clinical presentation and timely intervention likely contributed to the favorable outcome observed. Comparing this case to similar presentations can help overcome challenges and expedite diagnosis. Furthermore, the patient's understanding and commitment to medication compliance and lifestyle modifications are crucial for long-term success in managing his health.

Patient's perspective 

The patient expressed sincere gratitude towards all the specialists and the healthcare team involved in his care during his hospitalization. He eagerly anticipated returning home to reunite with and care for his granddaughter, acknowledging that such a reunion would not have been possible without the dedicated healthcare he received. 

## Conclusions

This case emphasizes the complex challenges involved in diagnosing and managing BCNE in the presence of pulmonary complications and implanted heart devices. It highlights the critical role of a collaborative healthcare approach, timely interventions, and patient education in achieving favorable outcomes. The importance of innovative interventions to address surgical complications and provide prompt, sustainable treatment is also highlighted. Additionally, this case highlights the importance of addressing psychosocial determinants early, especially in patients experiencing life stressors such as the loss of a loved one. Such stressors can lead to neglect of health and well-being, potentially resulting in similar clinical presentations.
